# Supplementary material for: Correlation between CD4 T-Cell Counts and Seroconversion among COVID-19 Vaccinated Patients with HIV: A Meta-Analysis
Source: Vaccines (Basel). 2023 Apr 4;11(4):789. doi: 10.3390/vaccines11040789 (PMC10141854; doi:10.3390/vaccines11040789)
Supplement: Supplementary file 1 [file vaccines-11-00789-s001.zip › vaccines-2204226-supplementary.pdf]

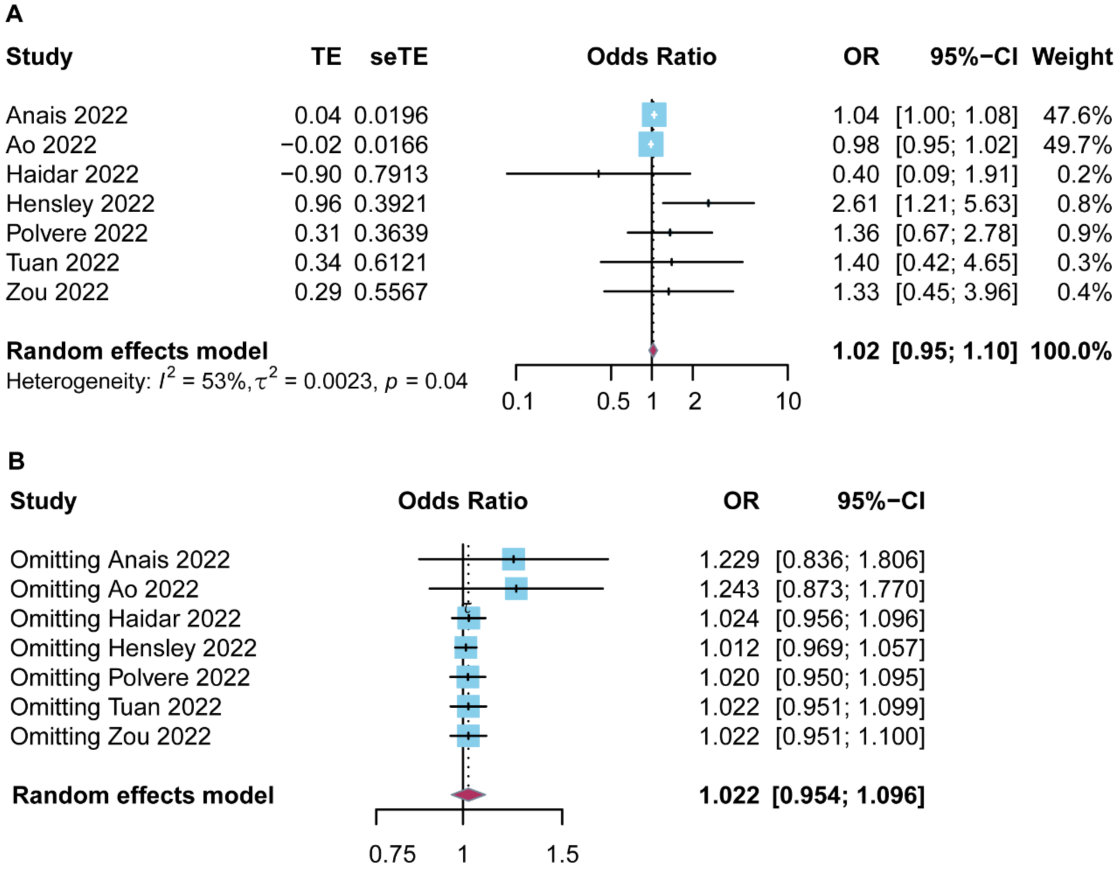

Figure S1. The forest plot (A), and the sensitivity analysis using "leaving-one-out" per time approach (B) for the pooled odds ratio of age associated with serologic response in patients with living HIV after COVID-19 vaccination.

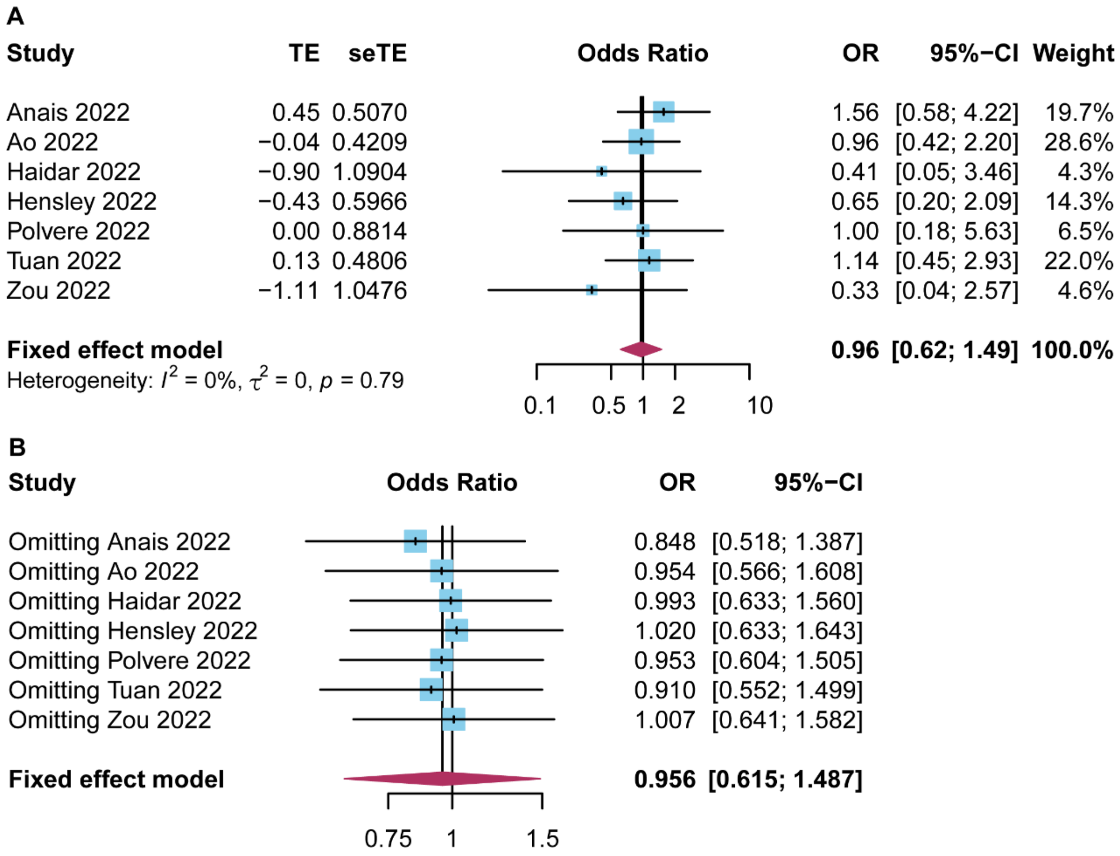

Figure S2. The forest plot (A), and the sensitivity analysis using "leaving-one-out" per time approach (B) for the pooled odds ratio of gender associated with serologic response in patients with living HIV after COVID-19 vaccination.

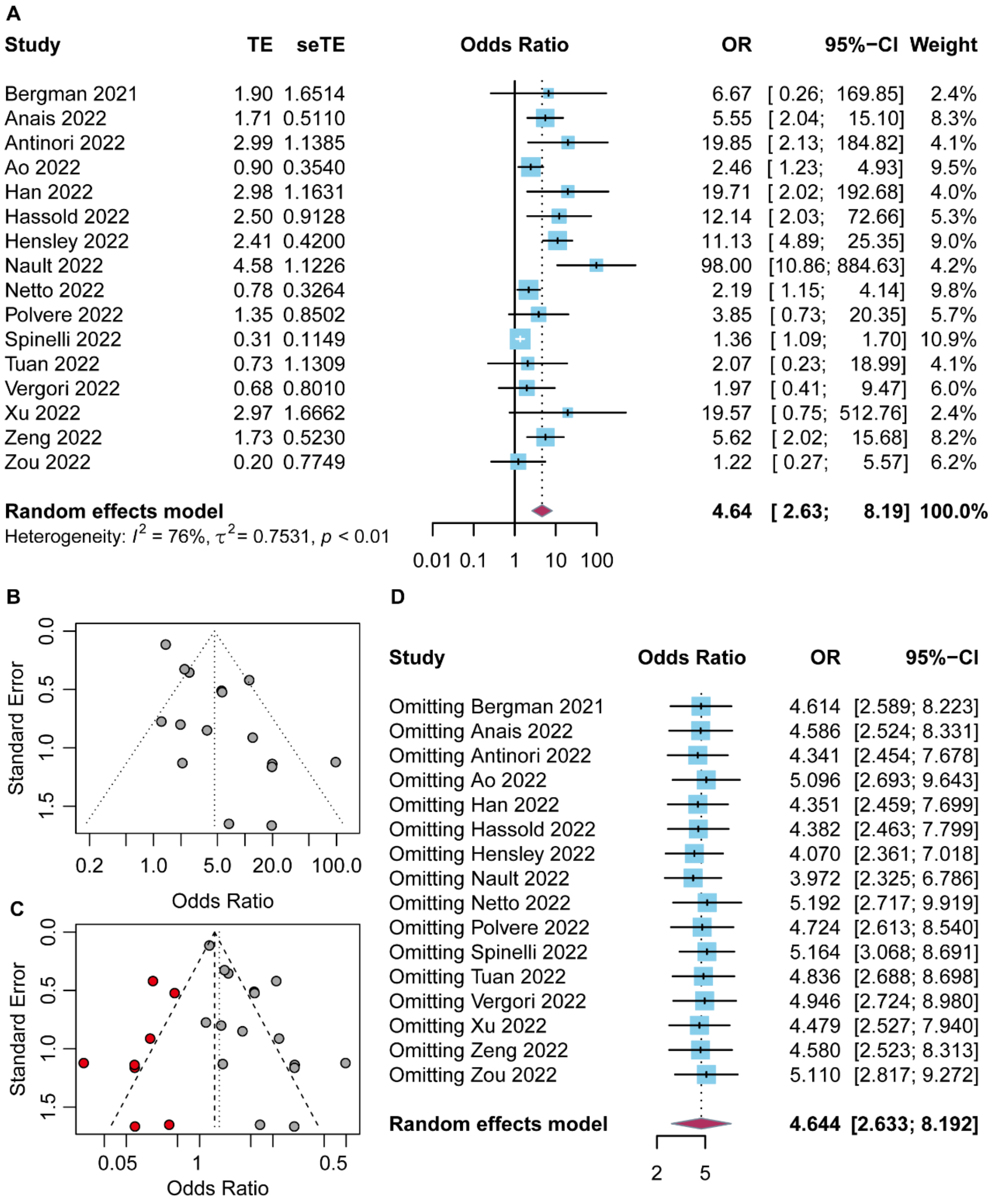

Figure S3. The forest plot (A), the funnel plot (B), the funnel plot after trim-and-fill analysis (C), and the sensitivity analysis using "leaving-one-out" per time approach (D) for the pooled odds ratio of CD4 T-cell counts associated with serologic response in patients with living HIV after COVID-19 vaccination.

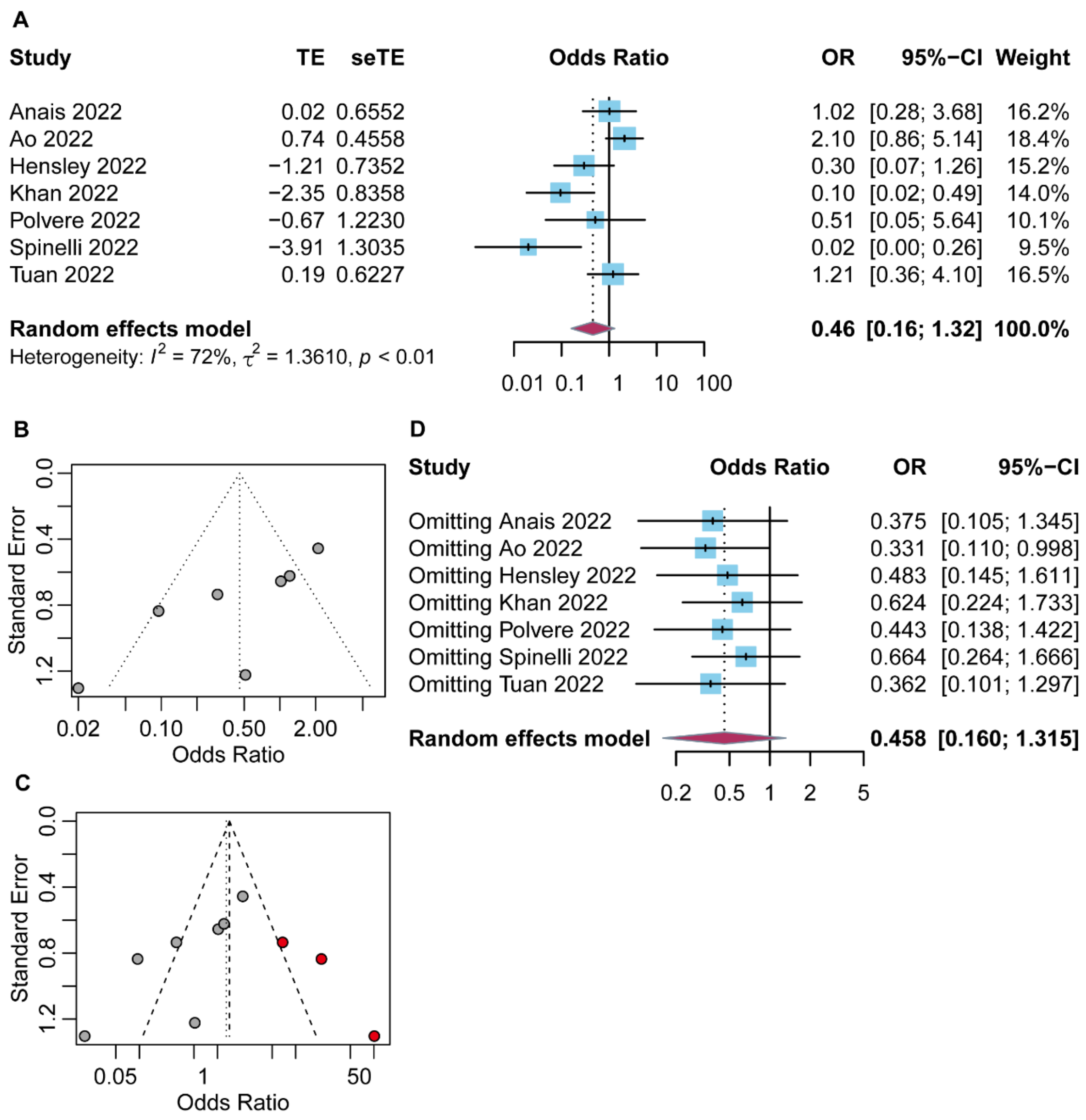

Figure S4. The forest plot (A), the funnel plot (B), the funnel plot after trim-and-fill analysis (C), and the sensitivity analysis using "leaving-one-out" per time approach (B) for the pooled odds ratio of HIV viral load associated with serologic response in patients with living HIV after COVID-19 vaccination.

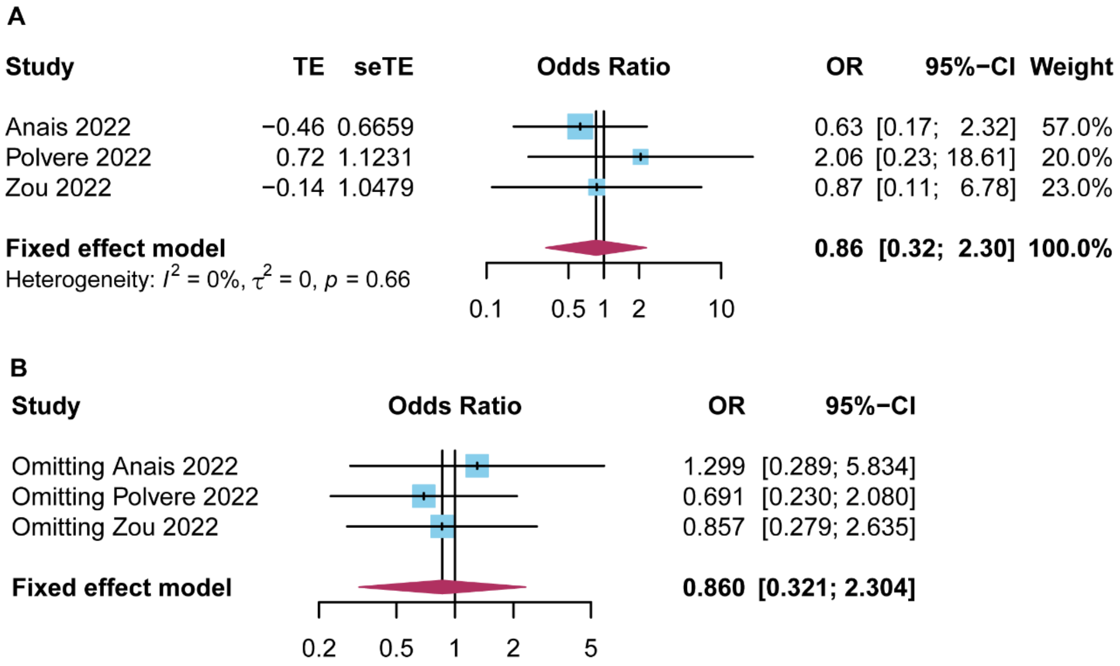

Figure S5. The forest plot (A), and the sensitivity analysis using "leaving-one-out" per time approach (B) for the pooled odds ratio of comorbidities associated with serologic response in patients with living HIV after COVID-19 vaccination.

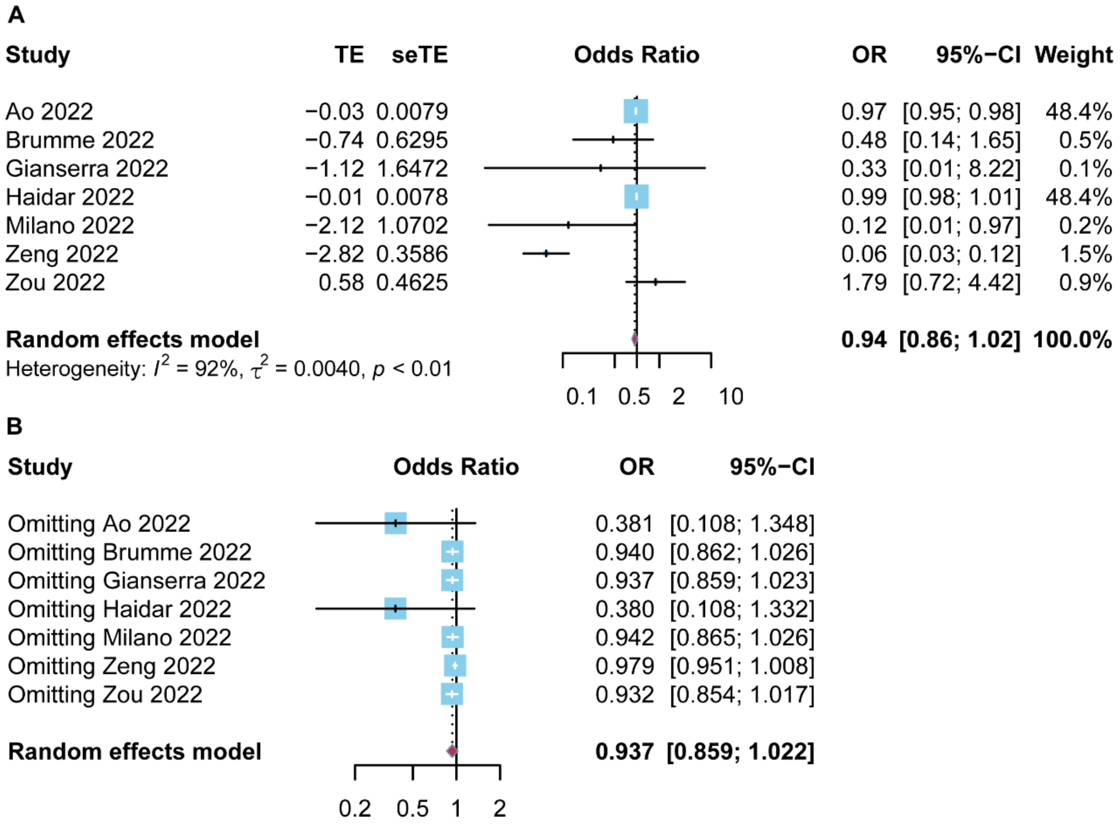

Figure S6. The forest plot (A), and the sensitivity analysis using "leaving-one-out" per time approach (B) for the pooled odds ratio of days after complete vaccination associated with serologic response in patients with living HIV after COVID-19 vaccination.

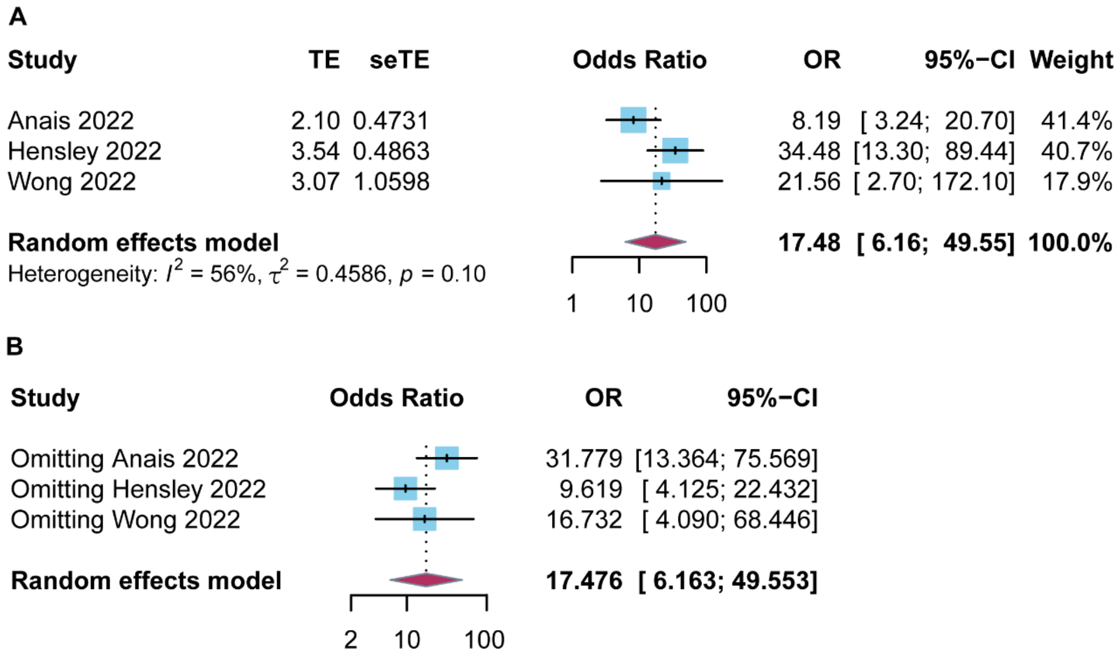

Figure S7. The forest plot (A), and the sensitivity analysis using "leaving-one-out" per time approach (B) for the pooled odds ratio of vaccine type associated with serologic response in patients with living HIV after COVID-19 vaccination.

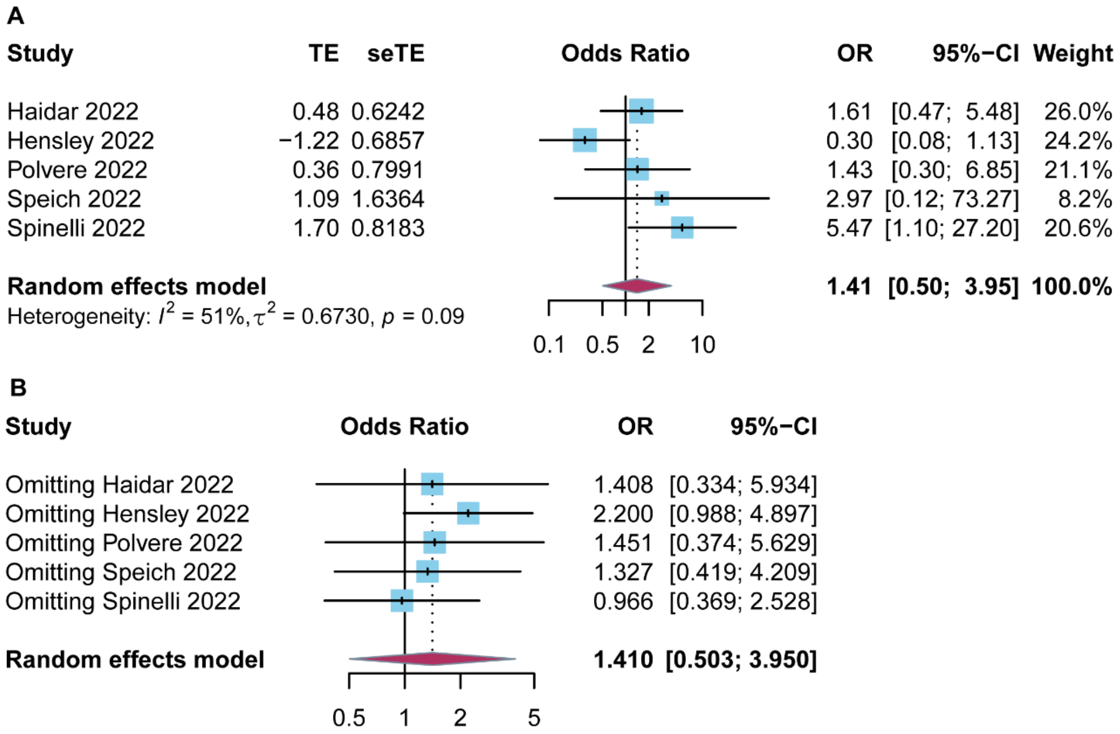

Figure S8. The forest plot (A), and the sensitivity analysis using "leaving-one-out" per time approach (B) for the pooled odds ratio of mRNA vaccine type associated with serologic response in patients with living HIV after COVID-19 vaccination.

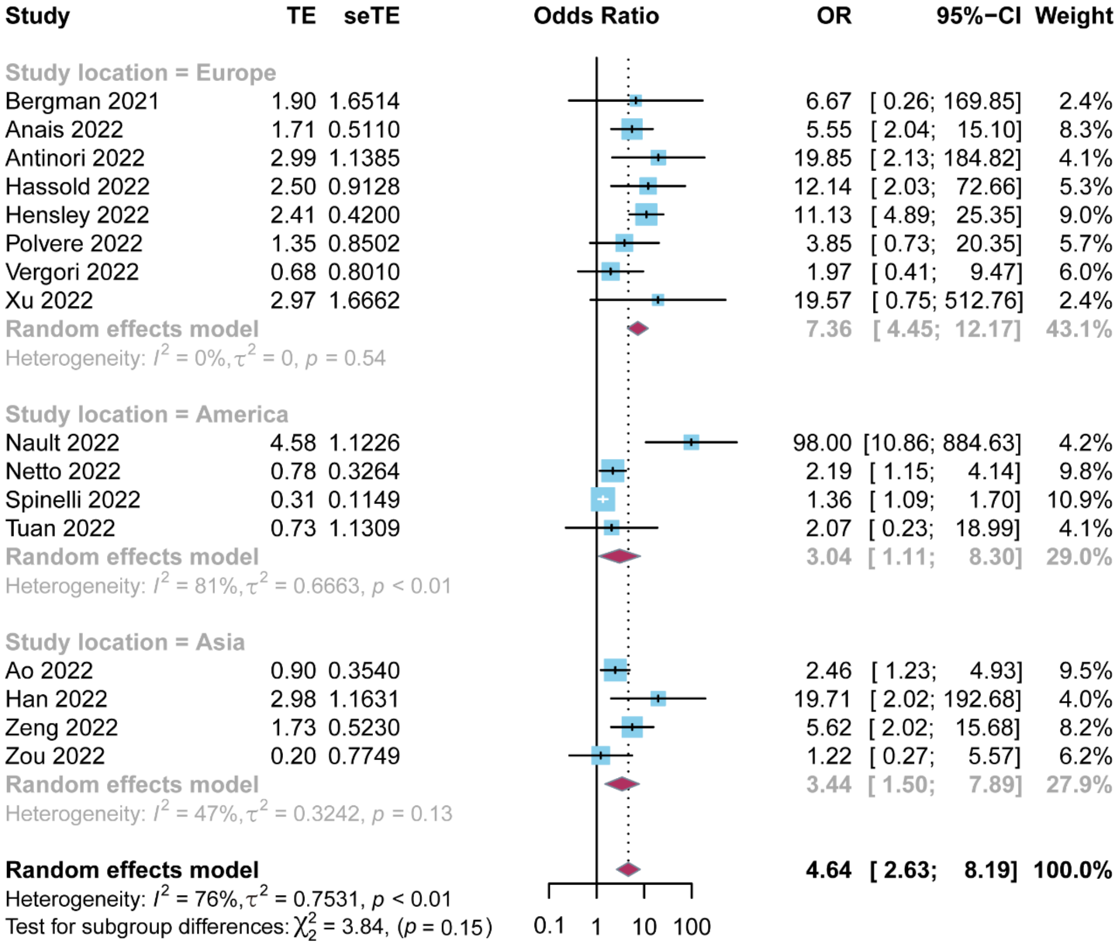

Figure S9. Subgroup analysis according to study location for the pooled odds ratio of CD4 counts associated with serologic response in patients with living HIV after COVID-19 vaccination.

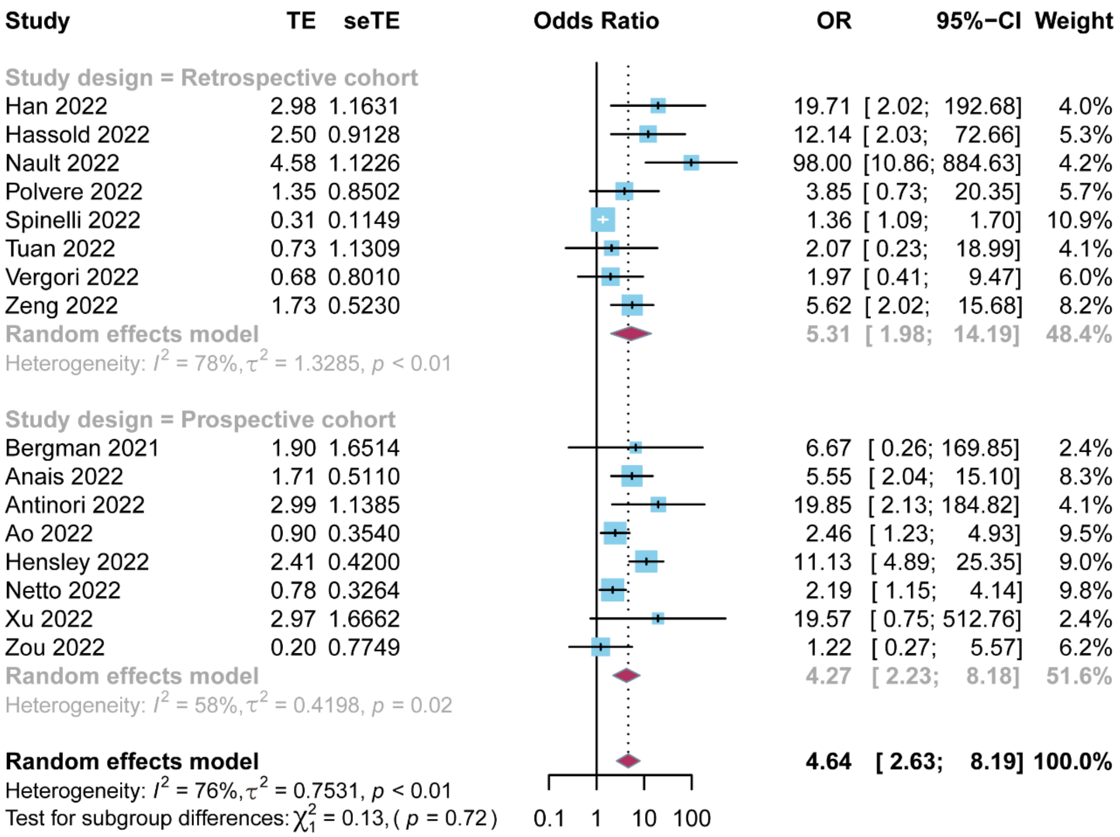

Figure S10. Subgroup analysis according to study design for the pooled odds ratio of CD4 counts associated with serologic response in patients with living HIV after COVID-19 vaccination.

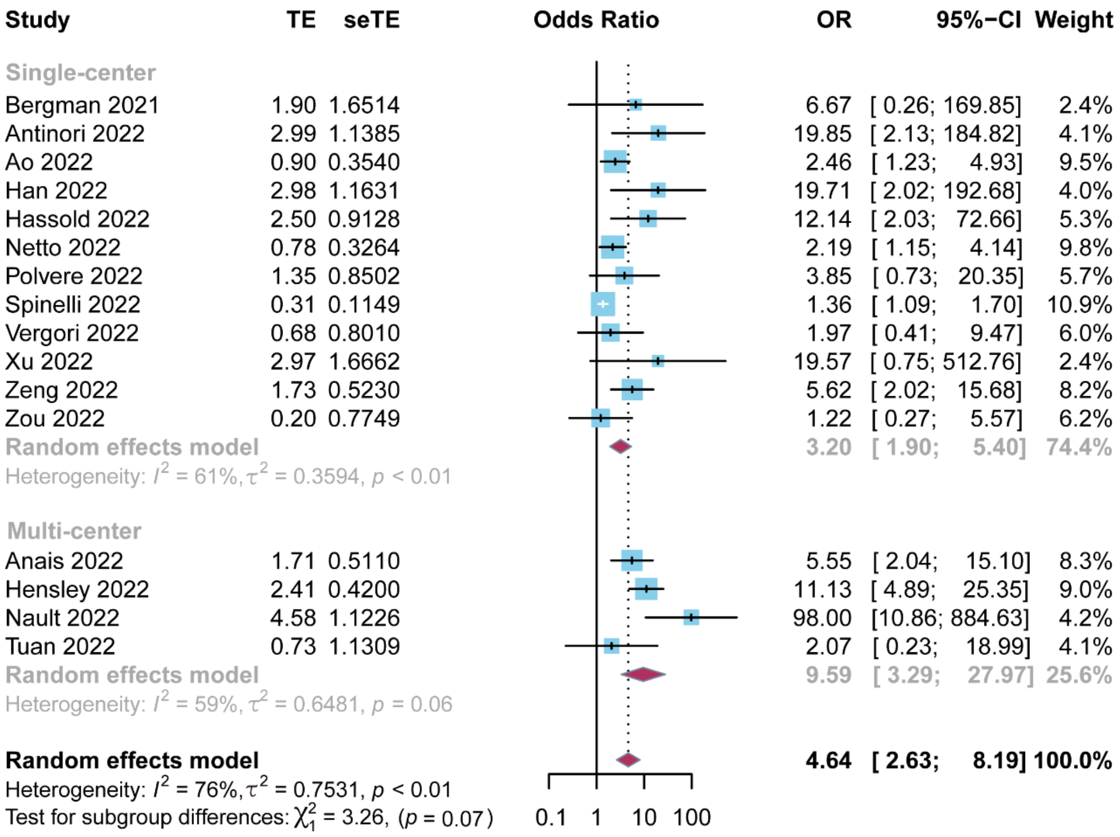

Figure S11. Subgroup analysis according to source of data for the pooled odds ratio of CD4 counts associated with serologic response in patients with living HIV after COVID-19 vaccination.

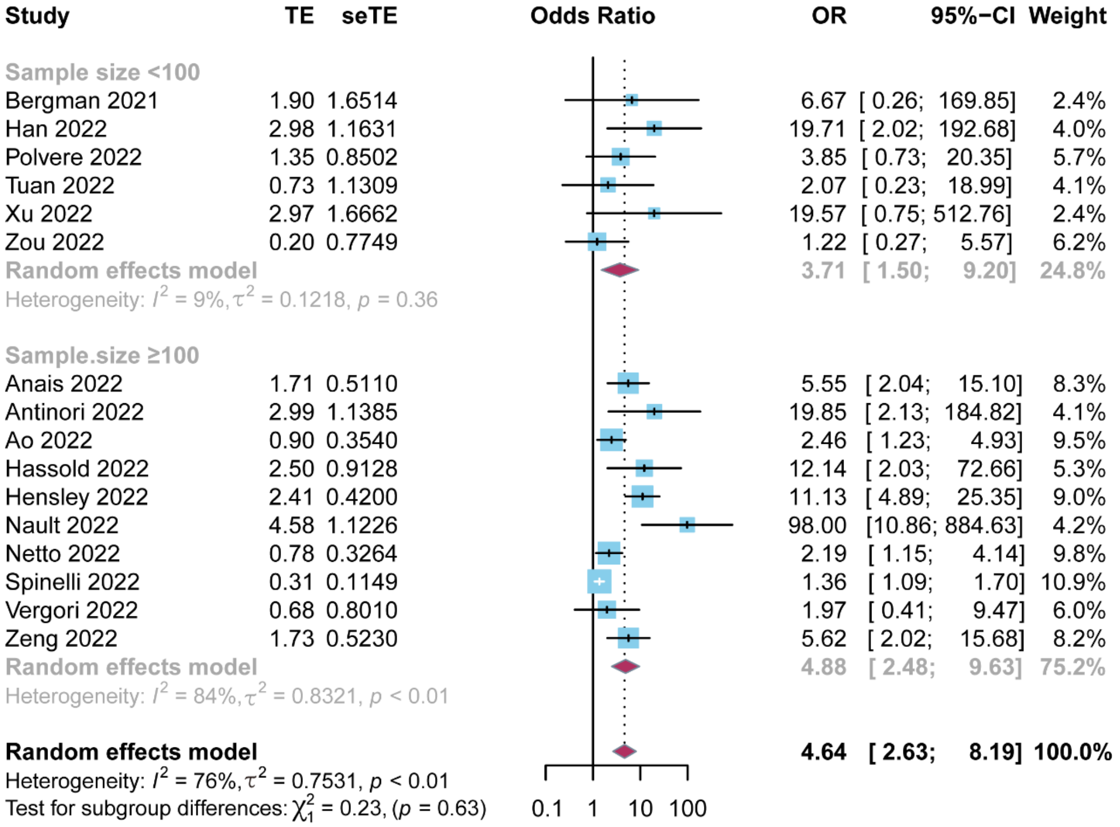

Figure S12. Subgroup analysis according to sample size for the pooled odds ratio of CD4 counts associated with serologic response in patients with living HIV after COVID-19 vaccination.

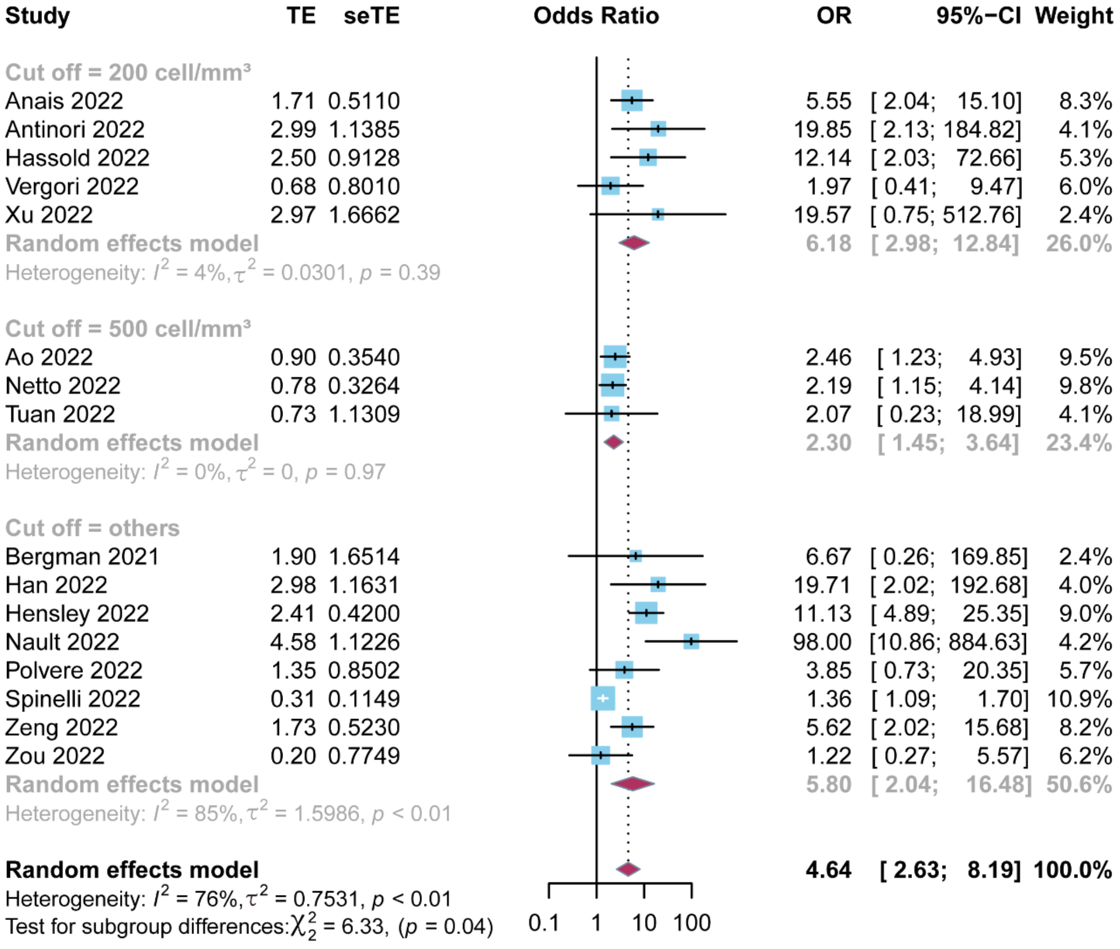

Figure S13. Subgroup analysis according to CD4 T-cell counts strata for the pooled odds ratio of CD4 counts associated with serologic response in patients with living HIV after COVID-19 vaccination.

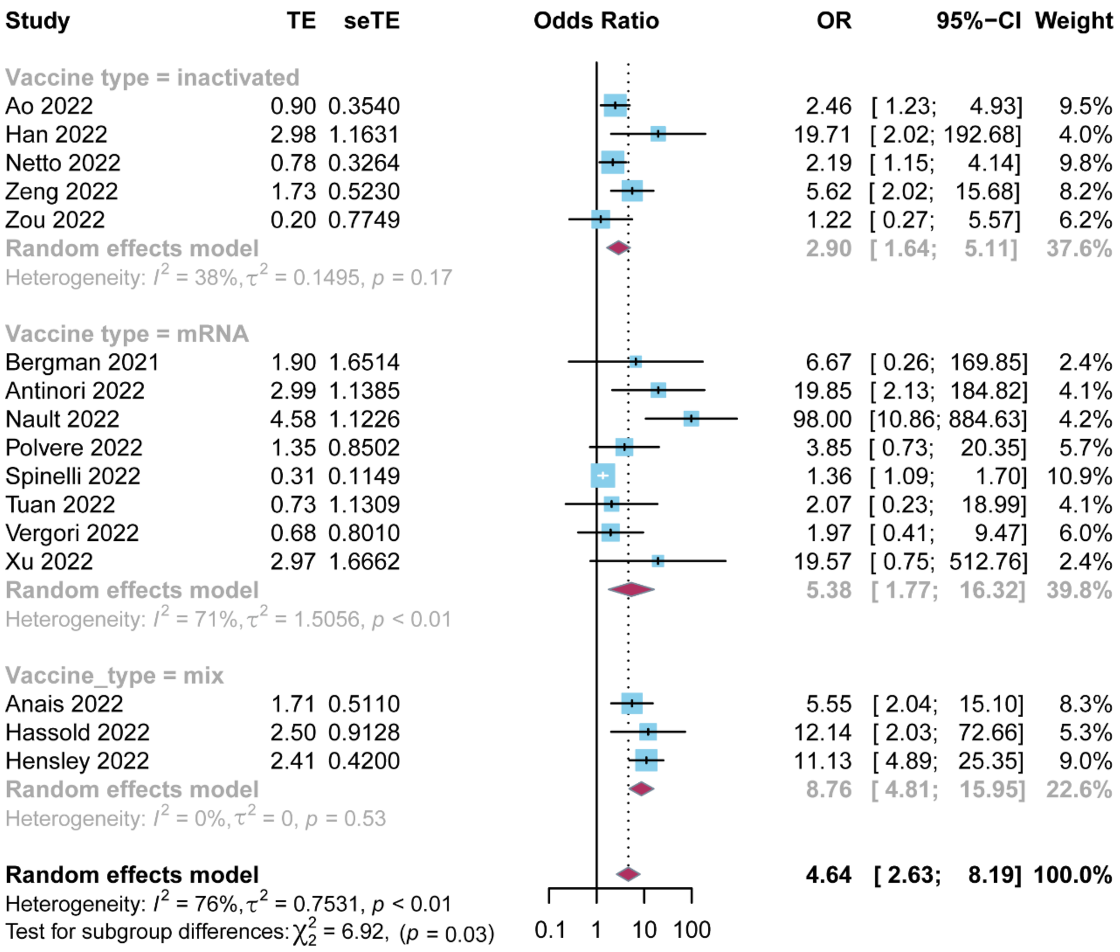

Figure S14. Subgroup analysis according to vaccine type for the pooled odds ratio of CD4 counts associated with serologic response in patients with living HIV after COVID-19 vaccination.

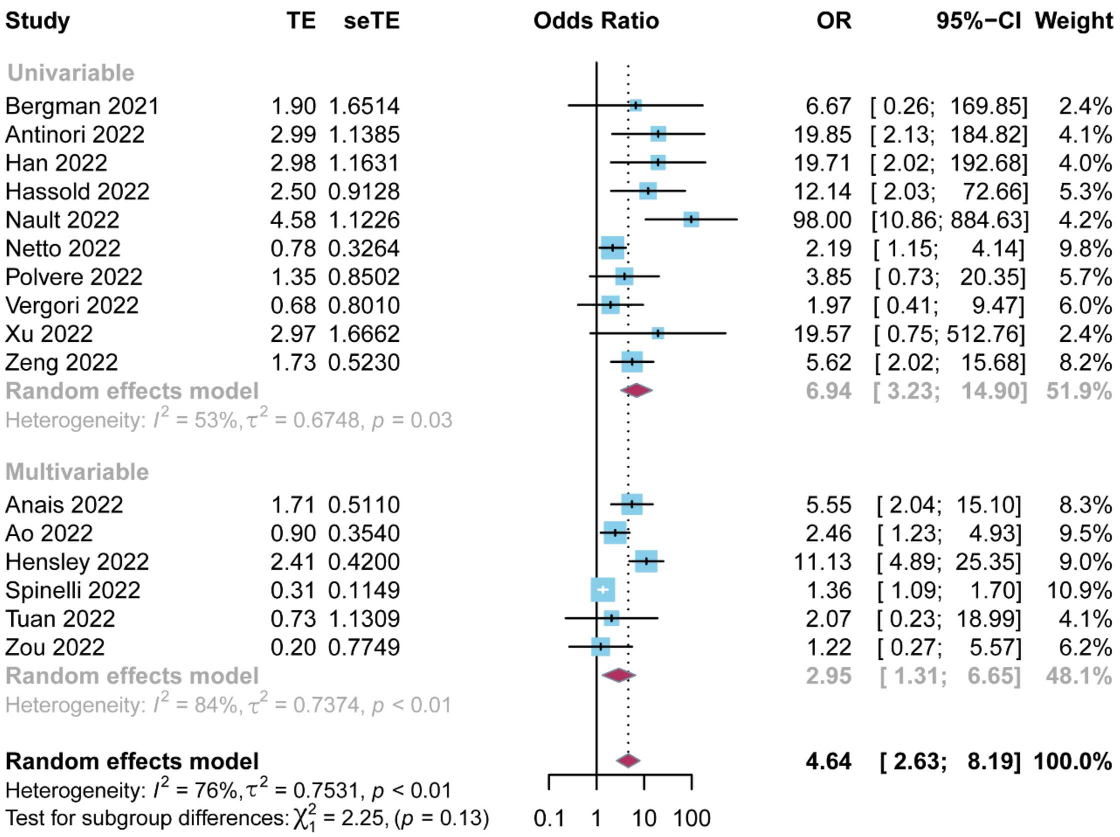

Figure S15. Subgroup analysis according to logistic regression analysis type for the pooled odds ratio of CD4 counts associated with serologic response in patients with living HIV after COVID-19 vaccination.

Table S1. The detailed search strategy.

Databases	Search Strategies	Articles' Number
PubMed	#1 corona[ti] OR covid*[ti] OR sars[ti] OR severe acute respiratory syndrome[ti] OR ncov*[ti] OR "severe acute respiratory syndrome coronavirus 2" [Supplementary Concept] OR "COVID-19" [Supplementary Concept] OR (wuhan[tiab] AND coronavirus[tiab]) OR (wuhan[tiab] AND pneumonia virus[tiab]) OR COVID19[tiab] OR COVID-19[tiab] OR coronavirus 2019[tiab] OR SARS-CoV-2[tiab] OR SARS2[tiab] OR SARS-2[tiab] OR "severe acute respiratory syndrome 2"[tiab] OR 2019-nCoV[tiab] OR (novel coronavirus[tiab] AND 2019[tiab]) NOT (animals[mesh] NOT humans[mesh])<br>#2 "Vaccines"[MeSH] OR "vaccination"[MeSH] OR vaccine[All Fields] OR vaccination[All Fields] OR vaccin*[All Fields]<br>#3 "HIV Infections" [MeSH] OR "HIV"[MeSH] OR "hiv"[tw] OR hiv infect*[tw] OR "human immunodeficiency virus"[tw] OR "human immunedeficiency virus"[tw] OR "human immuno-deficiency virus"[tw] OR "human immune-deficiency virus"[tw] OR ((human immun*) AND ("deficiency virus"[tw])) OR "acquired immunodeficiency syndrome"[tw] OR "acquired immunedeficiency syndrome"[tw] OR "acquired immuno-deficiency syndrome"[tw] OR "acquired immune-deficiency syndrome"[tw] OR ((acquired immun*) AND ("deficiency syndrome"[tw]))<br>#4 #1 AND #2 AND #3	708
Embase	#1 covid19 OR 'covid 19' OR 'sars cov 2' OR 'sars cov2' OR 'severe acute respiratory syndrome coronavirus 2' OR '2019 ncov' OR 2019ncov OR coronavirus<br>#2 'coronavirus infections'/exp<br>#3 'coronavirinae'/exp<br>#4 #1 OR #2 OR #3<br>#5 'vaccination'/exp OR vaccine OR vaccination OR vaccin*<br>#6 'human immunodeficiency virus infection'/exp OR 'human immunodeficiency virus'/exp OR 'hiv':ti,ab OR 'human immunodeficiency virus':ti,ab OR 'human immuno-deficiency virus':ti,ab OR 'human immunedeficiency virus':ti,ab OR 'human immune-deficiency virus':ti,ab OR 'acquired immune-deficiency syndrome':ti,ab OR 'acquired immunedeficiency syndrome':ti,ab OR 'acquired immunodeficiency syndrome':ti,ab OR 'acquired immuno-deficiency syndrome':ti,ab<br>#7 #4 AND #5 AND #6	603
Cochrane Library	#1 MeSH descriptor: [COVID-19] explode all trees<br>#2 covid-19 or covid19 or covid-19 or sars-cov-2 or sars-cov2 or 'severe acute respiratory syndrome coronavirus 2'or 2019ncov or coronavirus<br>#3 MeSH descriptor: [Coronaviridae] explode all trees<br>#4 #1 OR #2 OR #3<br>#5 MeSH descriptor: [HIV Infections] explode all trees<br>#6 MeSH descriptor: [HIV] explode all trees<br>#7 hiv OR 'hiv infect*' OR 'human immunodeficiency virus' OR 'human immunedeficiency virus' OR 'human immuno-deficiency virus' OR 'human immune-deficiency virus' OR ((human immun*) AND ("deficiency virus")) OR 'acquired immunodeficiency syndrome' OR 'acquired immunedeficiency syndrome' OR 'acquired immuno-deficiency syndrome' OR 'acquired immune-deficiency syndrome' OR ((acquired immun*) AND ('deficiency syndrome'))<br>#8 #5 OR #6 OR #7<br>#9 #4 AND #8	281
Duplications		-172
Total after duplications		1420

Table S2. Risk of bias of all included studies.

Risk of bias of randomized controlled trails (Cochrane Risk of Bias 2 Tool).

Studies (Year)	Sequence Generation	Allocation Conceal-ment	Blinding of Participants	Blinding of Outcome Assessors	Incomplete Data	Selective Outcomes Reporting	Other Sources	Overall Bias
Speich 2022	L	L	High	High	L	L	L	High

Risk of bias of cohort studies using the ROBINS-I scale.

Studies (Year)	Con-founding	Participant Selection	Classification of Interven-tions	Deviations from Intended Inter-ventions	Missing Data	Outcome Measure-ment	Selective Outcome Re-porting	Overall Bias
Bergman 2021	S	L	L	L	L	L	L	S
Antinori 2022	C	L	L	L	L	L	L	C
Ao 2022	C	L	L	L	L	L	L	C
Brumme 2022	S	L	L	L	L	L	L	S
Haidar 2022	C	L	L	L	L	L	L	C
Han 2022	S	L	L	L	L	L	L	S
Hensley 2022	C	L	L	L	L	L	L	C
Khan 2022	C	M	L	L	L	L	L	C
Nault 2022	C	M	L	L	L	L	L	C
Netto 2022	C	L	L	L	L	L	L	C
Polvere 2022	C	L	L	L	L	L	L	C
Spinelli 2022	M	L	L	L	L	L	L	M
Vergori 2022	C	L	L	L	L	L	L	C
Wong 2022	S	L	L	L	L	L	L	S
Xu 2022	C	L	L	L	L	L	L	C
Zeng 2022	C	L	L	L	L	L	L	C
Zou 2022	C	L	L	L	L	L	L	C

Table S3. Meta-regression for odds ratio for the seroconversion in COVID-19 vaccinated PLWH with high and low CD4 T cells.

Variable	β	95% LCI	95% UCI	*p*
Study location				
Europe	0.8601	-0.0755	1.7957	0.0716
America	-0.6694	-1.6967	0.3578	0.2015
Asia	-0.3624	-1.7000	0.9752	0.5954
Study design	-0.0927	-1.2635	1.0780	0.8766
Source of data	1.0619	-0.0085	2.1323	0.0519
Sample Size	0.1441	-1.1838	1.4720	0.8316
Cut off				
200 cell/mm^3	0.5634	-0.6888	1.8157	0.3779
500 cell/mm^3	-0.9765	-2.4818	0.5287	0.2035
Others	0.2503	-0.9855	1.4860	0.6914
Vaccine type				
Inactivated vaccine	-0.6323	-1.936	0.6717	0.3419
mRNA vaccine	-0.0130	-1.1538	1.1278	0.9822
mix	0.9181	-0.2190	2.0552	0.1135
Multivariable analysis	-0.8637	-1.9819	0.2545	0.1301

β：regression coefficient; LCI: lower confidence interval; UCI: upper confidence interval.

Table S4. Certainty of evidence and summary effect estimates assessed by GRADE (grading of recommendations, assessment, development, and evaluation) of the study outcomes. Meta-analysis of observational studies assessing factors associated with serological response to vaccine in people living with HIV.

Outcomes	Starting Level of Evidence	Quality assessment$					Reasons to Increase Level of Evidence*	Overall Quality of Evidence
		Risk of Bias	Inconsistency	Indirectness	Impression	Publication Bias		
Overall odds ratio of age associated with serological response to vaccination	Low	Serious	Serious	Not serious	Serious	Not serious	-	Very low
Overall odds ratio of gender associated with serological response to vaccination	Low	Serious	Not serious	Not serious	Serious	Serious	-	Very low
Overall odds ratio of CD4 count associated with serological response to vaccination	Low	Serious	Serious	Not serious	Serious	Serious	Dose-response gradient; Large effect	Very low
Overall odds ratio of HIV viral load associated with serological response to vaccination	Low	Serious	Serious	Not serious	Serious	Serious	-	Very low
Overall odds ratio of comorbidities associated with serological response to vaccination	Low	Serious	Not serious	Not serious	Serious	Not serious	-	Very low
Overall odds ratio of days after complete vacation associated with serological response to vaccination	Low	Serious	Serious	Not serious	Serious	Not serious	-	Very low
Overall odds ratio of vaccine type associated with serological response to vaccination	Low	Serious	Serious	Not serious	Serious	Not serious	Large effect	Very low
Overall odds ratio of mRNA vaccine type associated with serological response to vaccination	Low	Serious	Serious	Not serious	Serious	Not serious	-	Very low

$ Quality assessment to decrease level of evidence include risk of bias (≥25% of contributing studies were assessed as serious risk of bias), inconsistency (substantial between study heterogeneity, $I^2 \geq 50\%$), indirectness (presence of factors limiting generalizability of results), impression (95% confidence intervals for risk estimates are wide above 10% for outcomes), and publication bias (evidence of small study effects).* Reasons to increase level of evidence include large magnitude of effect (risk estimates less than 0.5), dose-response gradient or attenuation of the pooled risk estimates by plausible confounders.

File S1. List of excluded studies (n=40).

Failing to odds ratio of serologic response in HIV for COVID-19 vaccine (n = 33)

1. Chammartin F, Kusejko K, Pasin C, et al. Determinants of antibody response to severe acute respiratory syndrome coronavirus 2 mRNA vaccines in people with HIV. AIDS. 2022;36(10):1465-1468. doi:10.1097/QAD.0000000000003246
2. Healy K, Pin E, Chen P, et al. Salivary IgG to SARS-CoV-2 indicates seroconversion and correlates to serum neutralization in mRNA-vaccinated immunocompromised individuals. Med (N Y). 2022;3(2):137-153.e3. doi:10.1016/j.medj.2022.01.001
3. Woldemeskel BA, Karaba AH, Garliss CC, et al. Decay of coronavirus disease 2019 mRNA vaccine-induced immunity in people with HIV. AIDS. 2022;36(9):1315-1317. doi:10.1097/QAD.0000000000003263
4. Ogbe A, Pace M, Bittaye M, et al. Durability of ChAdOx1 nCoV-19 vaccination in people living with HIV. JCI Insight. 2022;7(7):e157031. Published 2022 Apr 8. doi:10.1172/jci.insight.157031
5. Chantasrisawad N, Puthanakit T, Tangsathapornpong A, et al. Immunogenicity and Reactogenicity of mRNA BNT162b2 COVID-19 Vaccine among Thai Adolescents with Chronic Diseases. Vaccines (Basel). 2022;10(6):871. Published 2022 May 29. doi:10.3390/vaccines10060871
6. Shinde V, Bhikha S, Hoosain Z, et al. Efficacy of NVX-CoV2373 Covid-19 Vaccine against the B.1.351 Variant. N Engl J Med. 2021;384(20):1899-1909. doi:10.1056/NEJMoa2103055
7. Gonzalez de Aledo M, Canizares A, Vazquez-Rodriguez P, et al. Safety and Immunogenicity of SARS-CoV-2 vaccines in people with HIV. AIDS 2022; 36(5): 691-5.
8. Balcells ME, Le Corre N, Duran J, et al. Reduced Immune Response to Inactivated Severe Acute Respiratory Syndrome Coronavirus 2 Vaccine in a Cohort of Immunocompromised Patients in Chile. *Clin Infect Dis* 2022; **75**(1): e594-e602.
9. BessenC, Plaza-Sirvent C, Bhat J, et al. Impact of SARS-CoV-2 vaccination on systemic immune responses in people living with HIV. 2022.
10. Chan DPC, Wong NS, Wong BCK, Chan JMC, Lee SS. Three-Dose Primary Series of Inactivated COVID-19 Vaccine for Persons Living with HIV, Hong Kong. Emerg Infect Dis 2022; 28(10): 2130-2.
11. Cossu MV, Mileto D, Giacomelli A, et al. Does the co-morbidity burden contribute to suboptimal immunological responses to COVID-19 vaccination in people living with HIV? J Infect Dis 2022.
12. Feng Y, Zhang Y, He Z, et al. Immunogenicity of an inactivated SARS-CoV-2 vaccine in people living with HIV-1: a non-randomized cohort study. EClinicalMedicine 2022; 43: 101226.
13. Frater J, Ewer KJ, Ogbe A, et al. Safety and immunogenicity of the ChAdOx1 nCoV-19 (AZD1222) vaccine against SARS-CoV-2 in HIV infection: a single-arm substudy of a phase 2/3 clinical trial. Lancet HIV 2021; 8(8): e474-e85.
14. Gidari A, Bastianelli S, Pierucci S, et al. BNT162b2 Elicited an Efficient Humoral Response Against Different Strains of SARS-CoV-2 in People Living with HIV. Curr HIV Res 2022; 20(4): 296-300.
15. Heftdal LD, Knudsen AD, Hamm SR, et al. Humoral response to two doses of BNT162b2 vaccination in people with HIV. J Intern Med 2022; 291(4): 513-8.
16. Jedicke N, Stankov MV, Cossmann A, et al. Humoral immune response following prime and boost BNT162b2 vaccination in people living with HIV on antiretroviral therapy. HIV Med 2022; 23(5): 558-63.
17. Lapointe HR, Mwimanzi F, Cheung PK, et al. People with HIV receiving suppressive antiretroviral therapy show typical antibody durability after dual COVID-19 vaccination, and strong third dose responses. medRxiv 2022.
18. Levy I, Wieder-Finesod A, Litchevsky V, et al. Immunogenicity and safety of the BNT162b2 mRNA COVID-19 vaccine in people living with HIV-1. Clin Microbiol Infect 2021; 27(12): 1851-5.
19. Lombardi A, Butta GM, Donnici L, et al. Anti-spike antibodies and neutralising antibody activity in people living with HIV vaccinated with COVID-19 mRNA-1273 vaccine: a prospective single-centre cohort study. Lancet Reg Health Eur 2022; 13: 100287.
20. Loubet P, Wittkop L, Ninove L, et al. One-month humoral response following two or three doses of messenger RNA coronavirus disease 2019 vaccines as primary vaccination in specific populations in France: first results from the Agence Nationale Recherche contre le Sida (ANRS)0001S COV-POPART cohort. Clin Microbiol Infect 2022.
21. Lv Z, Li Q, Feng Z, et al. Inactivated SARS-CoV-2 vaccines elicit immunogenicity and T-cell responses in people living with HIV. Int Immunopharmacol 2022; 102: 108383.
22. Madhi SA, Koen AL, Izu A, et al. Safety and immunogenicity of the ChAdOx1 nCoV-19 (AZD1222) vaccine against SARS-CoV-2 in people living with and without HIV in South Africa: an interim analysis of a randomised, double-

blind, placebo-controlled, phase 1B/2A trial. Lancet HIV 2021; 8(9): e568-e80.
23. Madhi SA, Moodley D, Hanley S, et al. Immunogenicity and safety of a SARS-CoV-2 recombinant spike protein nanoparticle vaccine in people living with and without HIV-1 infection: a randomised, controlled, phase 2A/2B trial. Lancet HIV 2022; 9(5): e309-e22.
24. Oyaert M, De Scheerder MA, Van Herrewege S, et al. Evaluation of Humoral and Cellular Responses in SARS-CoV-2 mRNA Vaccinated Immunocompromised Patients. Front Immunol 2022; 13: 858399.
25. Portillo V, Fedeli C, Ustero Alonso P, et al. Impact on HIV-1 RNA Levels and Antibody Responses Following SARS-CoV-2 Vaccination in HIV-Infected Individuals. Frontiers in Immunology 2022; 12.
26. Pourcher V, Belin L, Soulie C, et al. High seroconversion rate and SARS-CoV-2 Delta neutralization in people with HIV vaccinated with BNT162b2. AIDS 2022; 36(11): 1545-52.
27. Rahav G, Lustig Y, Lavee J, et al. BNT162b2 mRNA COVID-19 vaccination in immunocompromised patients: A prospective cohort study. EClinicalMedicine 2021; 41: 101158.
28. Ruddy JA, Boyarsky BJ, Bailey JR, et al. Safety and antibody response to two-dose SARS-CoV-2 messenger RNA vaccination in persons with HIV. AIDS 2021; 35(14): 2399-401.
29. Ruddy JA, Boyarsky BJ, Bailey JR, et al. Safety and antibody response to two-dose SARS-CoV-2 messenger RNA vaccination in persons with HIV. AIDS 2021; 35(14): 2399-401.
30. Schmidt KG, Harrer EG, Tascilar K, et al. Characterization of Serum and Mucosal SARS-CoV-2-Antibodies in HIV-1-Infected Subjects after BNT162b2 mRNA Vaccination or SARS-CoV-2 Infection. Viruses 2022; 14(3).
31. Tan Y, Zou S, Ming F, et al. Early Efficacy and Safety of the Third Dose Inactivated COVID-19 Vaccine Among People Living With HIV. J Acquir Immune Defic Syndr 2022; 90(3): e1-e3.
32. Tau L, Turner D, Adler A, et al. SARS-CoV-2 Humoral and Cellular Immune Responses of Patients With HIV After Vaccination With BNT162b2 mRNA COVID-19 Vaccine in the Tel-Aviv Medical Center. Open Forum Infect Dis 2022; 9(4): ofac089.
33. Woldemeskel BA, Karaba AH, Garliss CC, et al. The BNT162b2 mRNA Vaccine Elicits Robust Humoral and Cellular Immune Responses in People Living With Human Immunodeficiency Virus (HIV). *Clin Infect Dis* 2022; **74**(7): 1268-70.

Cross-sectional study (n = 5)

1. Cai S, Liao G, Yu T, et al. Immunogenicity and safety of an inactivated SARS-CoV-2 vaccine in people living with HIV: A cross-sectional study. J Med Virol. 2022;94(9):4224-4233. doi:10.1002/jmv.27872
2. Wu S, Zou S, Ming F, et al. Humoral Immune Response to Inactivated COVID-19 Vaccination at the 3rd Month among People Living with HIV. Preprint. Res Sq. 2022;rs.3.rs-1750225. Published 2022 Jun 27. doi:10.21203/rs.3.rs-1750225/v1
3. Huang X, Yan Y, Su B, et al. Comparing Immune Responses to Inactivated Vaccines against SARS-CoV-2 between People Living with HIV and HIV-Negative Individuals: A Cross-Sectional Study in China. Viruses. 2022;14(2):277. Published 2022 Jan 28. doi:10.3390/v14020277
4. Yan Y, Davgadorj C, Lyu C, Zhang S, Qiu Y. Immunogenicity of a third dose of inactivated COVID-19 vaccine in people living with HIV-1, HBV, and tuberculosis during the Omicron variant epidemic: A cross-sectional study. J Infect. 2022;85(4):e109-e111. doi:10.1016/j.jinf.2022.06.032
5. Liu Y, Han J, Li X, et al. COVID-19 Vaccination in People Living with HIV (PLWH) in China: A Cross Sectional Study of Vaccine Hesitancy, Safety, and Immunogenicity. Vaccines (Basel). 2021;9(12):1458. Published 2021 Dec 9. doi:10.3390/vaccines9121458

Review (n = 2)

1. Gianserra L, Donà MG, Giuliani E, et al. High seroconversion rate after vaccination with mRNA BNT162b2 vaccine against SARS-CoV-2 among people with HIV - but HIV viremia matters?. AIDS. 2022;36(9):1319-1320. doi:10.1097/QAD.0000000000003239
2. Wolday D, de Wit TFR. Inactivated SARS-CoV-2 vaccine for people with HIV. EClinicalMedicine.2022;45:101327. Published 2022 Mar 5.doi:10.1016/j.eclinm.2022.101327
